# *De novo* design of D–σ–A molecules as universal hosts for monochrome and white phosphorescent organic light-emitting diodes†
†Electronic supplementary information (ESI) available. See DOI: 10.1039/c8sc00282g


**DOI:** 10.1039/c8sc00282g

**Published:** 2018-03-08

**Authors:** Wen-Cheng Chen, Yi Yuan, Ze-Lin Zhu, Zuo-Quan Jiang, Shi-Jian Su, Liang-Sheng Liao, Chun-Sing Lee

**Affiliations:** a Center of Super-Diamond and Advanced Films (COSDAF) , Department of Chemistry , City University of Hong Kong , Hong Kong & City University of Hong Kong Shenzhen Research Institute , Shenzhen , Guangdong , PR China . Email: apcslee@cityu.edu.hk; b Jiangsu Key Laboratory for Carbon-Based Functional Materials & Devices , Institute of Functional Nano & Soft Materials (FUNSOM) , Collaborative Innovation Center of Suzhou Nano Science and Technology (Nano-CIC) , Soochow University , Suzhou , 215123 , PR China . Email: lsliao@suda.edu.cn; c State Key Laboratory of Luminescent Materials and Devices , Institute of Polymer Optoelectronic Materials and Devices , South China University of Technology , Guangzhou 510640 , PR China

## Abstract

Two novel D–σ–A host materials 11,11-bis(9-phenyl-9*H*-carbazol-3-yl)-11*H*-benzo[4,5]imidazo[1,2-*a*]indole (BII–BCz) and 4,4′-(11*H*-benzo[4,5]imidazo[1,2-*a*]indole-11,11-diyl)bis(*N*,*N*-diphenylaniline) (BII–TPA) are synthesized by using the sp^3^ carbon attached to a newly designed 11*H*-benzo[4,5]imidazo[1,2-*a*]indole building block to link two electron-donating groups. The resulting materials feature high triplet energy levels, good thermal properties and suitable photophysical properties as universal hosts for full-color phosphorescent organic light-emitting diodes (OLEDs). Both BII–BCz and BII–TPA reveal excellent performances in blue, green, yellow, orange and red phosphorescent OLEDs with the same device structure. The employment of the new D–σ–A materials as single hosts in white OLEDs with a simple device structure allows us to achieve state-of-the-art performances. A single-emissive-layer white device based on BII–BCz demonstrates the highest performance with an external quantum efficiency up to 28.2% at 1000 cd m^–2^. Furthermore, this device displays extremely stable emission spectra, with a ΔCIE of only (0.009, 0.005) from 1000 to 10 000 cd m^–2^.

## Abstract 2

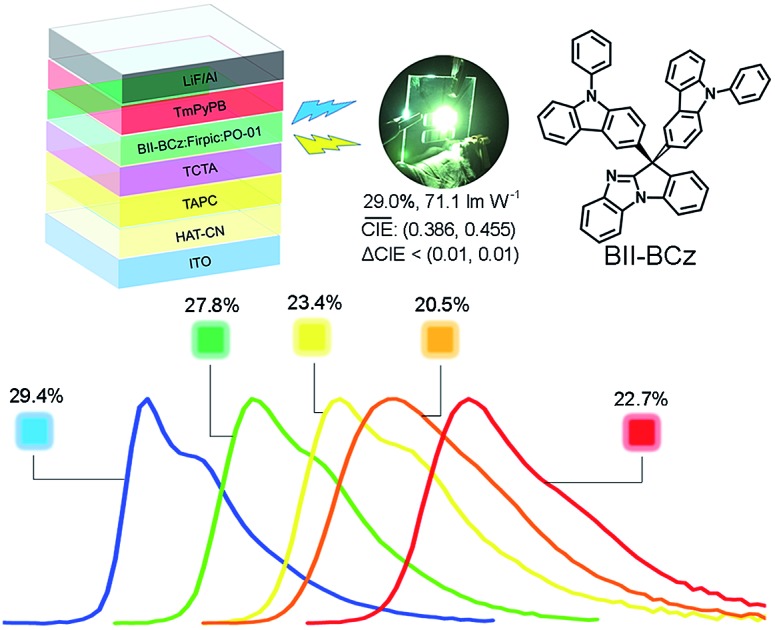
Novel D–σ–A molecules were developed as single hosts to achieve high performances in monochrome and white organic light-emitting diodes.

## Introduction

White organic light-emitting diodes (OLEDs) have been attracting much research interest stemming from their promising application in full-color flat-panel displays and large-area solid-state lightings.^1^ Due to the merits of the strong spin–orbit coupling effect, phosphorescent emitters can harness both singlet and triplet excitons to achieve 100% internal quantum efficiency^2^ and thus are widely used in white OLEDs.^3^–^7^ To minimize energy loss due to collision-induced quenching caused by the long emission lifetime of phosphors, they are typically dispersed in host materials with sufficiently high triplet energies (*E*_T_s) in most efficient phosphorescent OLEDs (PhOLEDs). In most cases, host molecules dominate (≥90 wt%) emissive layers (EMLs), and therefore have crucial influences on device performance, especially the driving voltage and power efficiency (PE).

With increasing demands on device performance, especially high efficiency at practical brightness (≥1000 cd m^–2^), much effort has been devoted to developing efficient host materials for all-phosphor white OLEDs.^8^–^15^ Reineke *et al.* reported very sophisticated device architectures in which two or more host materials with suitable frontier orbital energy levels are employed for respectively red, green and blue phosphors to decrease the carrier-transporting barrier, resulting in low driving voltages (∼3 and 4 V for 1000 and 10 000 cd m^–2^, respectively).^3^ Very recently, Wu and co-workers demonstrated power-efficient white OLEDs by using a blue exciplex to host blue and yellow phosphors.^16^ A low turn-on voltage (*V*_on_ = 2.5 V, at 1 cd m^–2^) and a high PE_max_ of 105 lm W^–1^ were realized due to the barrier-free carrier injection and transport properties of the bimolecular exciplex. However, it is difficult to optimize the device structure and precisely control the decomposition rate of the individual materials at the same time. Evidently, compared to the above-mentioned multi-host system, simplified devices employing a universal host deserve consideration in terms of repeatability and manufacturing cost. In this case, independent display pixels responsible for different color outputs can be fabricated with an analogous or even identical configuration, and single or multiple EMLs of lighting diodes can also be assembled expediently. However, to date, very few high-performance universal host materials for monochrome and white OLEDs are available in the literature.^17^–^19^ The scarcity of efficient universal hosts should be ascribed to the diverse requirements of multiple phosphorescent dopants, such as different frontier orbital levels, molecular polarities and host–guest interactions. The development of blue PhOLEDs, which are indispensable to generate white light emission, lagging behind those of green and red counterparts further raises the demand for host materials. Furthermore, to realize a high-performance lighting source, its white emission spectra must be stable over a wide range of brightness. Nevertheless, due to the intrinsic wide energy distribution, precise exciton allocation is difficult to execute ideally. To the best of our knowledge, there is so far no report on single-host-based white PhOLEDs that can simultaneously achieve external quantum efficiency (EQE) ≥25% at high brightness (≥1000 cd m^–2^) and stable electroluminescence (EL) emission with Commission Internationale de l'Éclairage (CIE) coordinate variation less than (0.01, 0.01). Therefore, seeking cost-effective, highly efficient single host materials as alternatives to multi-host systems has been a vibrant task for the development of white PhOLEDs.

Recently, the electron donor–π–acceptor (D–π–A) system demonstrated its versatility in the design of molecular host materials for RGB and white PhOLEDs, by virtue of its bipolar charge injection and transport properties.^17^–^23^ However, a D–π–A host often possesses enlarged conjugated extension due to the intramolecular charge transfer (ICT) interaction, resulting in *E*_T_ reduction. This would limit the application of D–π–A host materials in blue and white PhOLEDs. Therefore, until now few examples of highly efficient D–π–A type universal hosts for OLEDs are available in the literature. The D–σ–A molecular system is a feasible alternative to design bipolar universal hosts, because the σ-linking style can efficiently suppress ICT between the donor and the acceptor.^11^,^24^,^25^ However, most of the D–σ–A materials rely on fluorene to provide an sp^3^ carbon linking site. The directly conjugated fluorene fails to endow its derivatives with very high *E*_T_. Furthermore, in most cases, due to the electron neutrality of fluorene, it must be integrated with electron donating or withdrawing groups *via* its conjugated sites (C1–4), which may further lower *E*_T_.

**Fig. 1:**
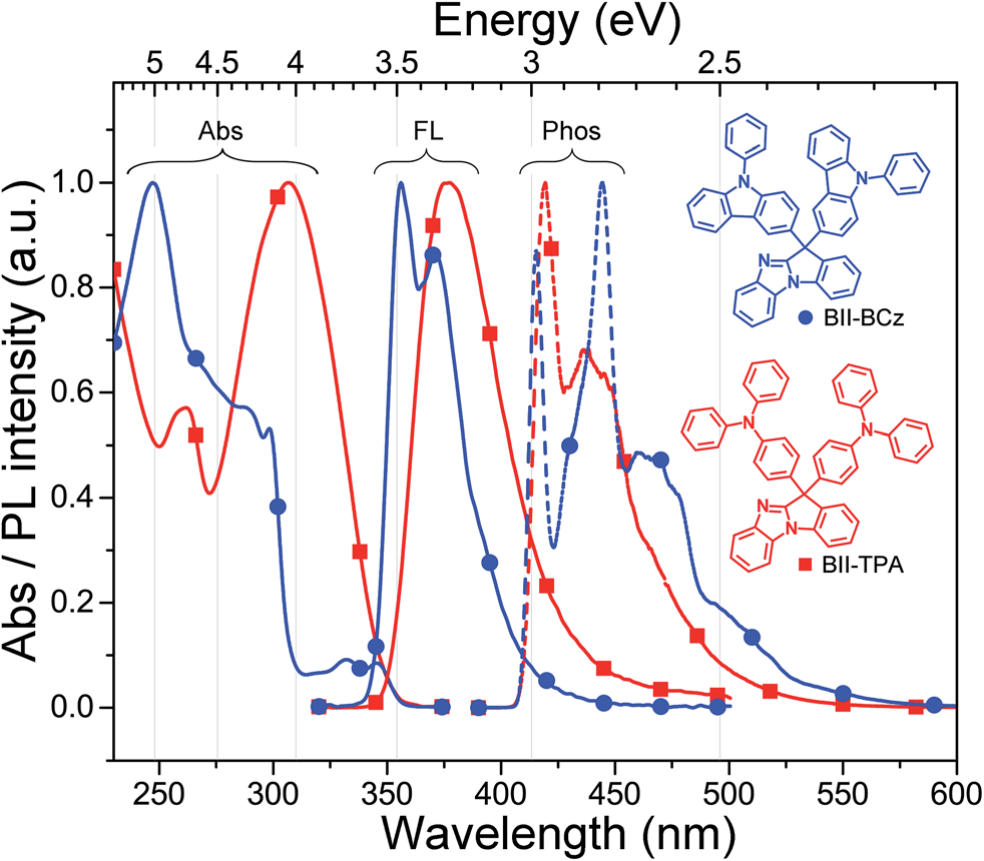
Absorption (Abs) and fluorescence (FL) spectra in CH_2_Cl_2_ at room temperature and phosphorescence (Phos) spectra in 2-methyltetrahydrofuran at 77 K of the new materials. Inset: chemical structures of BII–BCz and BII–TPA.

In this work, a new *n* type building block 11*H*-benzo[4,5]imidazo[1,2-*a*]indole (BII) is exploited for universal host materials. For the first time, we redesign the D–σ–A system, by using the inert sp^3^ carbon atom attached to the BII moiety to link two identical electron donor moieties—9-phenyl-9*H*-carbazole (BCz) or triphenylamine (TPA) groups—to construct two novel universal host materials, BII–BCz and BII–TPA, respectively (Fig. 1). BII consists of three parts: a benzo[*d*]imidazole (BI) group, a phenyl ring and an sp^3^ carbon. BII inherits electron-withdrawing properties from BI,^26^ while the phenyl ring attaches to BI *via* a non-conjugated C–N bond, which together makes BII an ideal electron acceptor with a high *E*_T_. The resulting materials BII–BCz and BII–TPA show high *E*_T_s of 2.98 and 2.95 eV, respectively, which surpass those of most of the fluorene-based host materials.^18^,^27^–^29^ BII–BCz and BII–TPA are suitable to host various phosphors, including FIrpic (blue), Ir(ppy)_2_(acac) (green), PO-01 (yellow), Ir(2-phq)_3_ (orange) and Ir(piq)_2_(acac) (red), with excellent performances. Monochrome devices employing BII–BCz as a host doped with phosphorescent emitters with a general device structure of ITO/HAT-CN (5 nm)/TAPC (50 nm)/TCTA (5 nm)/host:10 wt% dopant (20 nm)/TmPyPB (50 nm)/LiF (1 nm)/Al (100 nm) exhibit high EQEs of 29.4%, 27.8%, 23.4%, 20.5% and 22.7% for blue, green, yellow, orange and red diodes, respectively. Furthermore, by using BII–BCz as a single host, an all-phosphor white OLED featuring a single EML with a high EQE of 26.5% at a practical luminance of 5000 cd A^–1^ is also realized; meanwhile, this white PhOLED shows very stable EL spectra with a CIE variation of (0.009, 0.005) from 1000 to 10 000 cd m^–2^.

## Results and discussion

### Synthesis and characterization

BII–BCz and BII–TPA were synthesized *via* a facile two-step approach, as depicted in Scheme S1 in the ESI.† The cyclization reaction of 2-fluorobenzaldehyde with BI yielded the key precursor 11*H*-benzo[4,5]imidazo[1,2-*a*]indol-11-one (BIO) in an open atmosphere.^30^ An excessive amount of BCz or TPA was heated until completely melted as a solid solvent to dissolve BIO under N_2_ protection. Under the catalysis of methanesulfonic acid, the resulting mixture was heated to 170 °C for 10 h to afford BII–BCz or BII–TPA with good yields. Detailed synthetic procedures are given in the ESI.† BII–BCz and BII–TPA were characterized with ^1^H/^13^C NMR and mass analyses.

### Thermal properties

The thermal properties of BII–BCz and BII–TPA were studied with thermal gravimetric analysis (TGA) and differential scanning calorimetry (DSC) at a heating rate of 10 °C min^–1^ under N_2_, (Fig. S1†), and the key data are listed in Table 1. The two host materials exhibit high decomposition temperatures (*T*_d_s, 5% weight loss) over 400 °C and high glass transition temperatures (*T*_g_s) of 177 °C (BII–BCz) and 133 °C (BII–TPA). BII–BCz shows better thermal properties than BII–TPA, which can be attributed to the high rigidity of carbazole. These high *T*_d_ and *T*_g_ values indicate that the new materials are thermally stable during device fabrication *via* vacuum evaporation and capable of resisting Joule heat during operation.

**Table 1:** Summary of the physical data of BII–BCz and BII–TPA

Compd	*T*_d_ ^*a*^ (°C)	*T*_g_ ^*b*^ (°C)	HOMO^*c*^ (eV)	LUMO^*d*^ (eV)	*E*_g_ ^*e*^/*E*_T_^*f*^ eV	*λ*_abs_ (nm)		*λ*_fl_ (nm)	
						Solution	Film	Solution	Film
BII–BCz	438	177	–5.70	–2.26	3.44/2.98	247, 286, 298, 332, 334	248, 298	356, 371	362, 375
BII–TPA	412	133	–5.34	–1.90	3.44/2.95	261, 307	263, 313	378	370

^*a*^5% weight loss temperature.

^*b*^Glass transition temperature.

^*c*^Measured by cyclic voltammetry.

^*d*^Calculated from LUMO = HOMO + *E*_g_.

^*e*^Estimated from the absorption onset in films.

^*f*^Measured in 2-methyltetrahydrofuran at 77 K.

### Photophysical properties

The absorption and fluorescence spectra of BII–BCz and BII–TPA in CH_2_Cl_2_, and phosphorescence spectra in 2-MeTHF at 77 K are illustrated in Fig. 1. Both BII derivatives show the n–π* electronic transition of BI peaking around 300 nm.^31^ The characteristic absorption bands of carbazole derivatives are observed in BII–BCz at 286 nm (^1^L_a_ ← ^1^A transition, strong) and ∼330 nm (^1^L_b_ ← ^1^A transition, weak),^32^,^33^ while the strong absorption band at 247 nm can be ascribed to the π–π* transition. A small peak at 261 nm in the BII–TPA absorption profile, which is not found in BII–BCz, can be attributed to the n–π* transition of the TPA moiety. The absorption spectra in films are homologous to those in solutions (Fig. S2†). Optical energy gaps estimated from the absorption onset measured in films are ∼3.44 eV for both materials. Compared with BII–TPA, BII–BCz emits shorter-wavelength fluorescence with dual peaks both in solutions and films. The fluorescence profiles of BII–TPA and BII–BCz are similar to those of their donor moieties with no evidence of ICT properties.^33^,^34^ We also measured the fluorescence spectra of BII–BCz and BII–TPA in solutions with different polarities (Fig. S3†). The two materials show much less redshifts (<15 nm) compared with those (> 50 nm) of BI integrated BCz^35^ or TPA^36^ D–π–A molecules in the literature. This further suggests that ICT is efficiently suppressed by virtue of the D–σ–A configuration. As a result, high *E*_T_s of 2.98 and 2.95 eV for BII–BCz and BII–TPA are obtained, respectively, which are calculated from the vibronic 0–0 transition of phosphorescence spectra at 77 K. These high *E*_T_ values can sustain positive triplet energy transfer to full-color phosphorescent dopants.

### Theoretical calculations

**Fig. 2:**
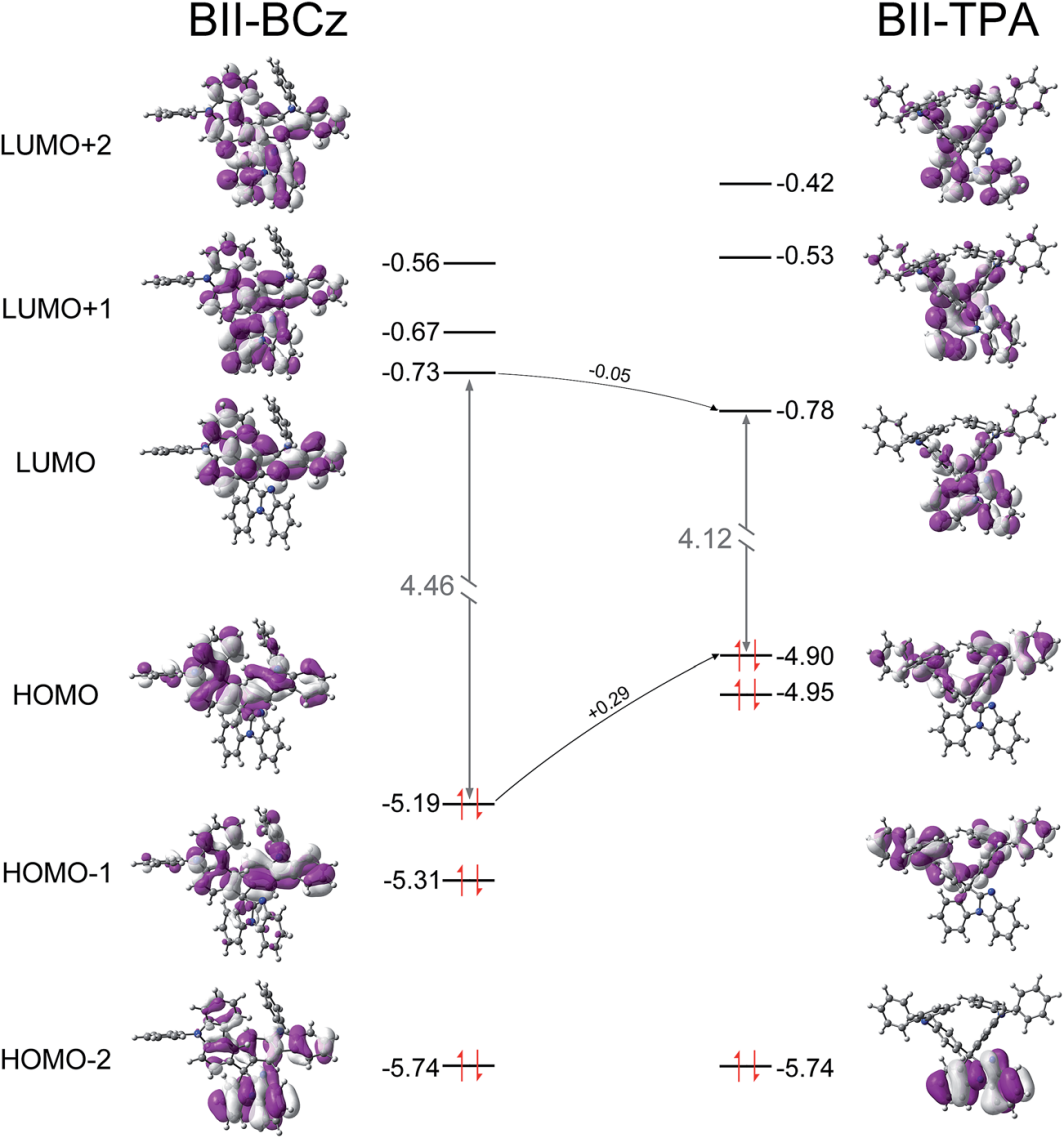
Spatial distributions of the molecular orbitals in BII–BCz and BII–TPA.

To further understand the structure–property relationship, the quantum chemical properties of BII–BCz and BII–TPA were studied by using time-dependent density functional theory (TD-DFT) at the B3LYP/6-31g(d,p) level. Fig. S4† shows the energy-minimized molecular configurations of the new compounds. The BII backbone exhibits high planarity similar to the case of fluorene. The pendent BCz and TPA moieties warp the BII scaffold, providing BII–BCz and BII–TPA with bulky molecular spacing. These 3D configurations may be partly responsible for the high *T*_g_ values, and also favor the decrease of structural-relaxation-induced exciton quenching.^37^ Spatial distributions of the molecular orbitals are shown in Fig. 2. The highest occupied molecular orbital (HOMO) and the lowest unoccupied molecular orbital (LUMO) of BII–TPA are respectively localized on the TPA and the BII segments. The two BCz groups in BII–BCz dominate the HOMO distribution as expected. Meanwhile, the carbazole contains a directly electronically coupled biphenyl, enabling the LUMO electron cloud to also concentrate on the segments near the biphenyl in BII–BCz. In consideration of the other unoccupied molecular orbitals, such as the LUMO+1 (–0.56 eV) and LUMO+2 (–0.67 eV), with narrow energy gaps compared to the LUMO (–0.73 eV) and localized distribution on the BII moiety, the BII in BII–BCz would have a contribution to electron injection and transportation. It is worth noting that the HOMO level of BII–TPA shows an increment of 0.29 eV compared to that of BII–BCz, while their LUMO levels remain unchanged, revealing the better hole injection ability of BII–TPA.

### Monochrome devices

**Fig. 3:**
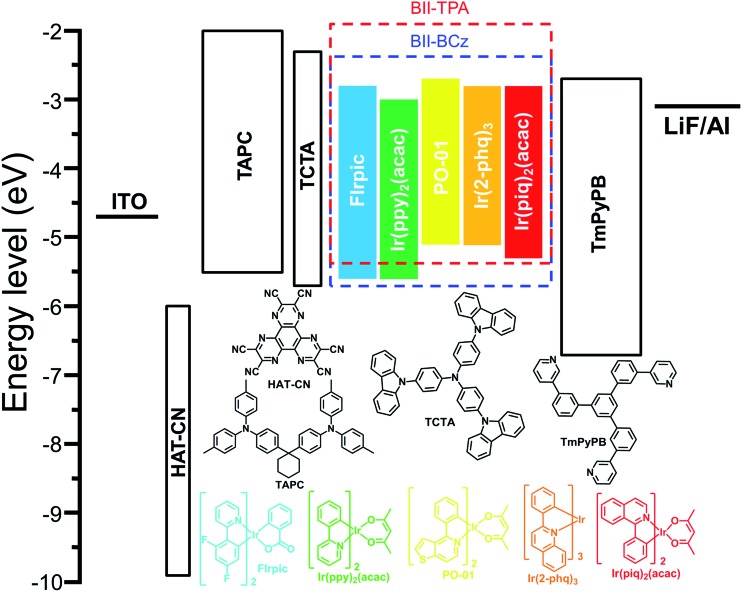
Device structures and the energy levels of the materials used in the devices.

Five phosphorescent emitters, FIrpic (blue), Ir(ppy)_2_(acac) (green), PO-01 (yellow), Ir(2-phq)_3_ (orange) and Ir(piq)_2_(acac) (red), with different emission colors, are selected as dopants to evaluate the performance of BII–BCz and BII–TPA as host materials. Fig. S5† shows the absorption spectra of the phosphors and the photoluminescence (PL) spectra of the hosts in CH_2_Cl_2_. Good overlaps between the absorption of phosphors and the emission of hosts suggest that exothermic energy transfer from hosts to dopants can be realized in OLEDs. Before device fabrication, the HOMO levels of the host materials are determined with cyclic voltammetry (CV) to be –5.70 and –5.34 eV for BII–BCz and BII–TPA, respectively (Fig. S6†). Combined with the *E*_g_ values, the LUMO levels are estimated to be –2.26 and –1.90 eV, respectively (the LUMO levels estimated from CV reduction scans are also provided in Fig. S6† for reference). We first fabricated a set of PhOLEDs with a general structure of ITO/HAT-CN (5 cm)/TAPC (50 nm)/TCTA (5 nm)/host:10 wt% dopant (20 nm)/TmPyPB (50 nm)/LiF (1 nm)/Al (100 nm), where the host is BII–BCz or BII–TPA, HAT-CN is the hole injection layer, TAPC is the hole transport layer, TCTA is the buffer layer, TmPyPB^38^ is the electron transport layer and LiF is the electron injection layer. Fig. 3 shows the chemical structures of the materials used in the devices, as well as their corresponding energy levels. Key device data are summarized in Table 2.

**Table 2:** Key performance data of the BII–BCz and the BII–TPA based PhOLEDs

Device	Host	Voltage^*a*^ (V)	CIE^*b*^ (*x*, *y*)	CE^*c*^ (cd A^–1^)	PE^*c*^ (lm W^–1^)	EQE^*c*^ (%)
B1	BII–BCz	3.1, 3.6, 4.2	(0.16, 0.33)	59.1, 57.2	53.0, 48.6	29.4, 28.2
B2	BII–TPA	3.1, 3.6, 4.2	(0.17, 0.34)	45.2, 35.7	40.6, 26.2	21.2, 16.5
G1	BII–BCz	2.9, 3.3, 3.8	(0.35, 0.61)	95.2, 92.9	96.2, 76.1	27.8, 25.8
G2	BII–TPA	2.8, 3.2, 3.7	(0.34, 0.62)	96.3, 94.4	94.9, 76.7	26.4, 25.9
Y1	BII–BCz	2.9, 3.6, 4.4	(0.53, 0.47)	59.3, 57.2	50.6, 41.1	23.4, 22.6
Y2	BII–TPA	2.6, 3.1, 3.6	(0.52, 0.48)	80.3, 77.6	79.9, 63.1	29.7, 28.0
O1	BII–BCz	3.4, 4.8, 6.5	(0.58, 0.41)	38.1, 29.7	34.2, 15.6	20.5, 15.6
O2	BII–TPA	3.4, 4.2, 5.3	(0.58, 0.42)	33.4, 30.2	28.3, 18.9	17.1, 15.9
R1	BII–BCz	3.3, 4.6, 6.8	(0.68, 0.31)	15.8, 8.9	16.5, 4.1	22.7, 13.4
R2	BII–TPA	2.8, 3.4, 4.8	(0.68, 0.31)	15.0, 12.1	15.1, 7.6	22.1, 17.9
WD1	BII–BCz	3.3, 3.7, 4.2	(0.30, 0.42)	52.0, 51.6	40.9, 38.2	20.3, 20.2
WD2	BII–BCz	3.2, 3.6, 4.1	(0.33, 0.43)	65.5, 65.1	53.0, 47.9	25.0, 24.6
WD3	BII–BCz	3.2, 3.6, 4.1	(0.34, 0.44)	66.6, 66.1	53.5, 47.6	25.2, 25.0
WD4	BII–TPA	3.2, 3.8, 4.7	(0.23, 0.37)	53.2, 48.4	44.1, 32.3	23.7, 21.2
WD5	BII–TPA	3.2, 3.7, 4.6	(0.29, 0.40)	56.7, 54.1	45.2, 36.7	23.6, 21.1
WD6	BII–TPA	3.2, 3.7, 4.7	(0.30, 0.40)	49.5, 47.1	40.0, 30.9	20.0, 18.6
WS	BII–BCz	3.1, 3.6, 4.2	(0.39, 0.46)	81.6, 79.1	71.1, 58.2	29.0, 28.2

^*a*^Voltage at 1, 100, and 1000 cd m^–2^, respectively.

^*b*^Measured at 1000 cd m^–2^.

^*c*^Device performances corresponding to the value at maximum and 1000 cd m^–2^, respectively.

**Fig. 4:**
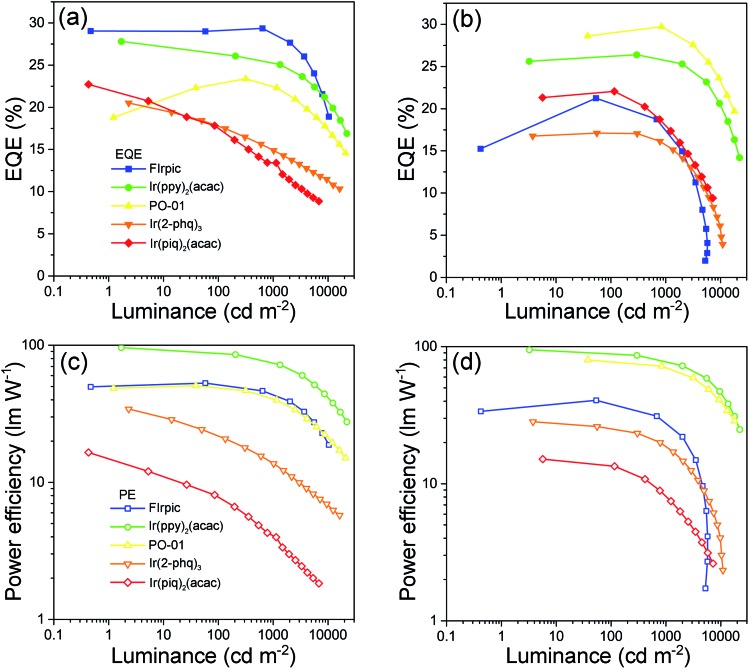
Performances of the monochrome PhOLEDs: EQE–luminance curves of (a) the BII–BCz and (b) the BII–TPA based devices; PE–luminance curves of (c) the BII–BCz and (d) the BII–TPA based devices.

As aforementioned, high-performance blue PhOLEDs are difficult to realize due to the high *E*_T_s of blue dopants. In our case, BII–BCz and BII–TPA have sufficiently high *E*_T_s approaching 3.0 eV, which are competent to confine triplet excitons within the blue phosphor FIrpic (*E*_T_ = 2.62 eV). Fig. S7† shows the EL spectra of the BII–BCz (solid line) and the BII–TPA (dashed line) based blue devices (denoted as device B1 and B2, respectively). Both devices show characteristic FIrpic EL emissions, with CIE coordinates of (0.16, 0.33) and (0.17, 0.34), respectively. As shown in Fig. 4 and Table 2, the maximum EQEs for devices B1 and B2 are 29.4% and 21.2%, respectively. Note that device B1 retains a high EQE of 28.2% and a power efficiency (PE) of 48.6 lm W^–1^ at a luminance of 1000 cd m^–2^, proving to be one of the most efficient FIrpic-based devices.^16^,^39^–^42^ By contrast, device B2 shows serious efficiency roll-off at luminance higher than 1000 cd m^–2^. To figure out the possible mechanism, transient decay PL spectra are recorded on host:10 wt% FIrpic doped films (30 nm), as shown in Fig. S8.† The BII–TPA based film demonstrates a longer PL decay, showing a delay component of 0.66 μs. It is suggested that the concentration of triplet excitons in the EML of device B2 is much higher than that of device B1, leading to a higher probability of triplet–triplet annihilation (TTA) for serious efficiency roll-off.

Next, we examined the device performance of the new hosts using low energy dopants. Fig. S9† shows the current density–voltage–luminance (*J*–*V*–*L*) characteristics of the monochrome devices. The green devices hosted by BII–BCz (device G1) and BII–TPA (device G2) show low *V*_on_s of <3 V and excellent performances. Devices G1 and G2 exhibit maximum efficiencies of 95.2 cd A^–1^, 96.2 lm W^–1^, and 27.8%, and 96.3 cd A^–1^, 94.9 lm W^–1^, and 26.4%, respectively, with slow efficiency roll-offs. Interestingly, for the PO-01 based yellow OLEDs, the device hosted by BII–TPA (device Y2) demonstrates superior performances with an EQE_max_ (PE_max_) of 29.7% (79.9 lm W^–1^), outperforming those of the BII–BCz based counterpart (device Y1, 23.4% and 50.6 lm W^–1^). In fact, it has been pointed out that the PO-01 dopant can act as an electron trap in the host–guest system.^43^,^44^ In our case, a larger LUMO gap of BII–TPA/PO-01 than that of BII–BCz/PO-01 and a small gap of PO-01/TmPyPB indicate that electrons can be directly injected into the PO-01 dopant in device Y2 more easily. From the *J*–*V* curves of their single carrier-only devices of the hosts (Fig. S10†), BII–TPA is much more hole dominating. The use of the PO-01 dopant may provide another electron transport channel and thus balance the carrier flow in the EML, leading to higher efficiencies in device Y2. The varying EL spectral widths also indicate that the charge migration and recombination zone may be changed upon using different dopants (Fig. S7†). Two types of devices, B1 and B2, and G1 and G2, show identical EL spectra with the same device structures. On the other hand, due to the weak microcavity effect, the devices based on BII–TPA with longer wavelength emissions (the yellow, orange and red devices) show narrower EL bandwidths compared to those of BII–BCz, which implies that, after traps are filled up, recombination zones would be changed by another electron transport channel (PO-01) in the BII–TPA based devices. The new materials also successfully host the Ir(2-phq)_3_ (orange) and Ir(piq)_2_(acac) (red) phosphors to realize decent EL performances, as shown in Fig. 4 and Table 2. For example, devices O1 and O2 achieve 20.5% and 17.1% EQEs at maximum, and produce orange EL peaks at around 590 nm with CIE coordinates of (0.58, 0.41) and (0.58, 0.42), respectively. Similar excellent performances are also achieved in red devices R1 and R2. Both of them exhibit highly efficient deep red EL emissions with CIE coordinates of (0.68, 0.31) and EQEs of 22.7% and 22.1% for R1 and R2, respectively. It is worth noting that R2 gives slow efficiency roll-off and low driving voltages. At 1000 cd m^–2^, a high EQE of 17.9% can be retained. Overall, these device performances are among the best for PhOLEDs employing a universal host, and even comparable to those of the most advanced monochrome PhOLEDs.

### White devices

**Fig. 5:**
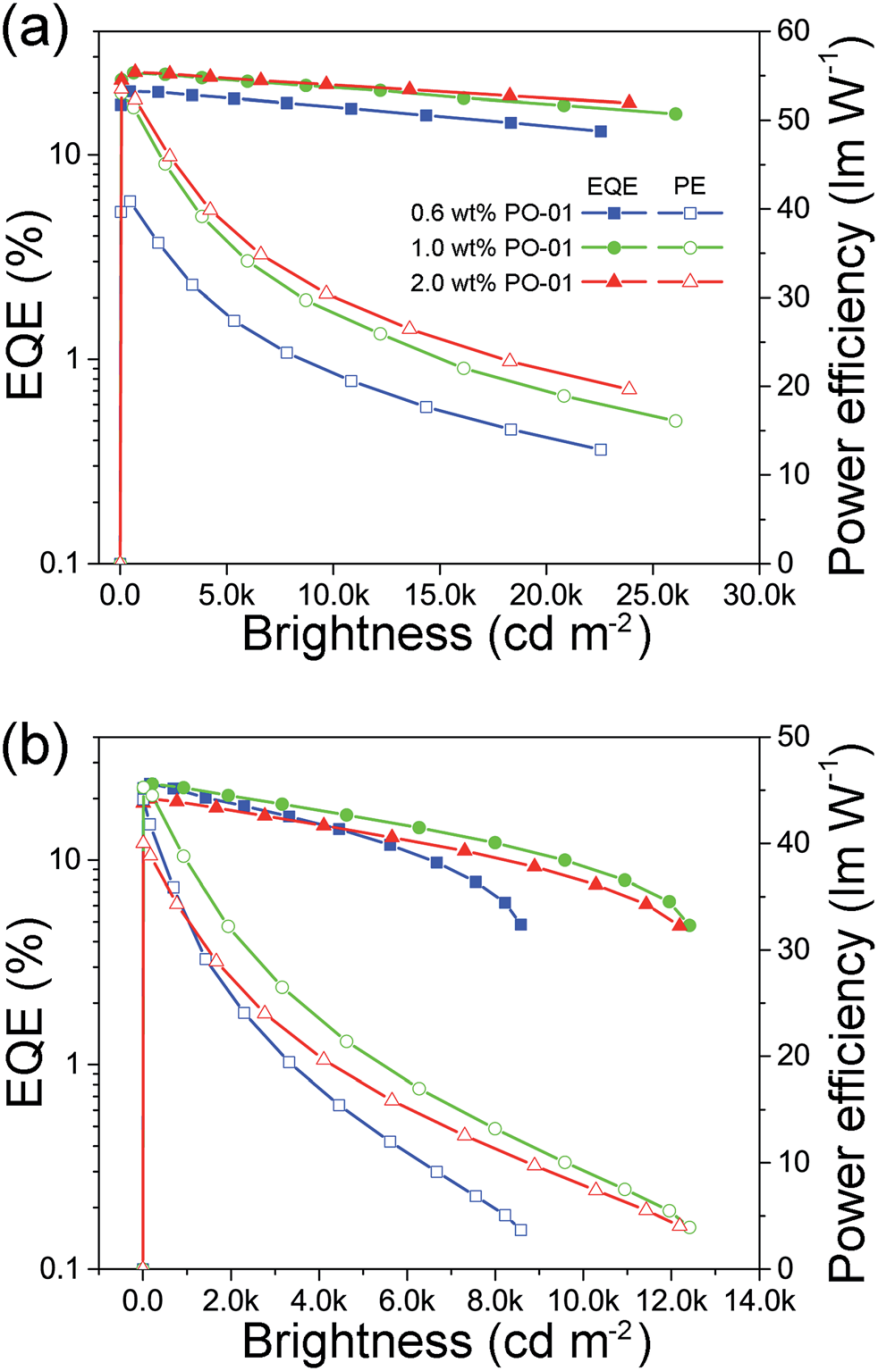
EQE–brightness–PE curves of the dual-EML white devices based on (a) BII–BCz and (b) BII–TPA.

Given the superior performances of monochrome PhOLEDs, white PhOLEDs were fabricated by using BII–BCz and BII–TPA as a single host. Herein, we mixed blue and yellow light, two complementary colors, to generate white light emission conveniently. First, white PhOLEDs employing a dual-EML (D-EML) configuration were fabricated with a general structure of ITO/HAT-CN (5 cm)/TAPC (50 nm)/TCTA (5 nm)/host:10 wt% FIrpic (19 nm)/host:*x* PO-01 (1 nm)/TmPyPB (50 nm)/LiF (1 nm)/Al (100 nm) (*x* = 0.6, 1.0 and 2.0 wt%; host = BII–BCz (device WD1–3) or BII–TPA (device WD4–6)). In this D-EML strategy, a thin PO-01 layer is sandwiched between the FIrpic and the TmPyPB layers; therefore PO-01 can capture excitons *via* downhill transfer from the FIrpic layer. The device performances are shown in Fig. 5 and Table 2. The *V*_on_s of the D-EML white device are ∼3.2 V, almost the same as those of the corresponding blue devices, indicating that the insertion of the yellow layers does not significantly affect the charge injection and transportation. The performances are roughly proportional to the concentration of the PO-01. Device WD3 shows the best performances with an EQE_max_ up to 25.2% and a decent PE_max_ of 53.5 lm W^–1^. Note that although the maximum efficiencies of the BII–TPA based devices (WD4–6) are comparable to those of devices employing TPA–BII as the host (WD1–3), devices WD4–6 show more obvious efficiency roll-offs at high brightness. For example, device WD3 demonstrates a superior EQE of 22.0% at 10 000 cd m^–2^, while the corresponding figure for device WD6 is only 7% (drops from 20.0% at maximum). This can be attributed to TTA quenching from the BII–TPA hosted FIrpic layers.

Because the yellow EML is very thin and energy transfer from the FIrpic layer to the PO-01 layer can only happen near the interface, most of the D-EML devices in this study exhibit cold white EL emissions. White EL devices involve complicated exciton distribution, and changes in the emission spectra under different biases can provide information on the charge and exciton behavior in the devices. The voltage-dependent EL spectra of the D-EML white OLEDs are shown in Fig. S11 (WD1–3) and S12 (WD4–6).† Fig. S11d and S12d† depict that the yellow component of the white emission first increases and then decreases when voltage increases progressively. As discussed above, PO-01 can act as a trap in the host–guest system. In the initial low bias region (slightly higher than the *V*_on_), the electrons injected from the TmPyPB layers will fill up the PO-01 trap sites preferentially, leading to less exciton recombination in the FIrpic layers. Upon further bias increases (around 4 to 4.5 V), the trap sites are filled up completely, resulting in maximum yellow light intensity. The larger spectral change in the low bias region in BII–TPA based devices also supports this point, because the trap effect is more evident when BII–TPA is used as the host. In the high bias region, electron injection becomes more efficient and more excitons recombine in the FIrpic layer, leading to reduction in yellow light intensity. This effect becomes more significant on increasing the PO-01 concentration, because the trap number rises as well. This non-monotonic spectral change can help to restrain the color shift of the white devices. For instance, device WD3 has similar white color CIE coordinates of (0.34, 0.44), (0.34, 0.44) and (0.34, 0.43) at a luminance of 1000, 5000 and 10 000 cd m^–2^, respectively.

**Fig. 6:**
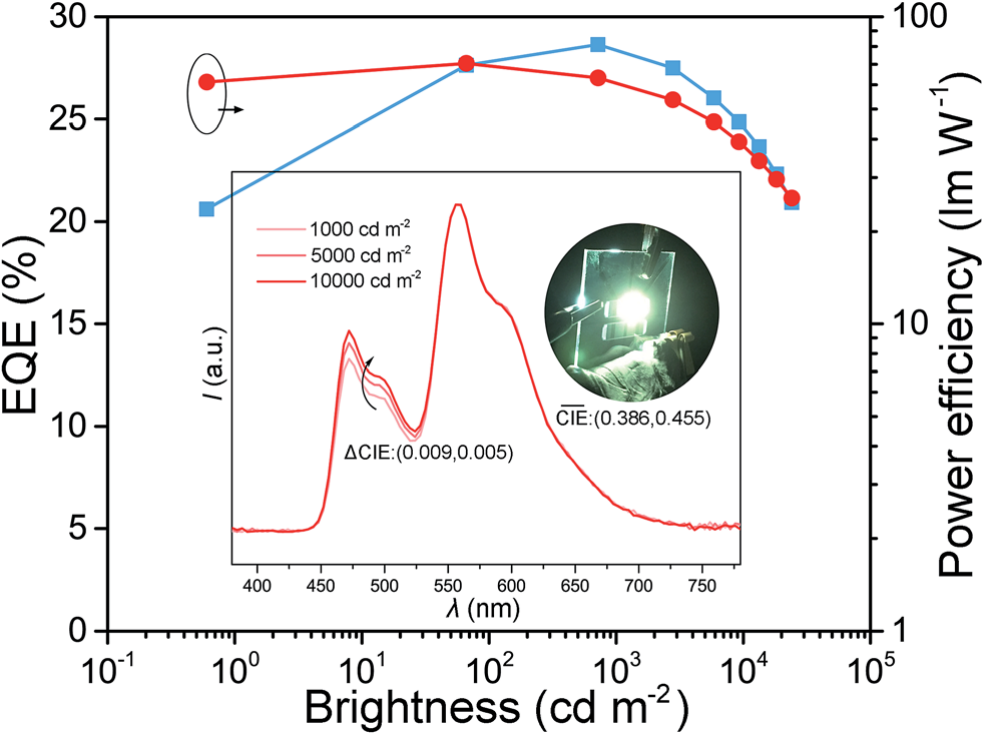
EQE–brightness–PE curves of the single-EML white device based on BII–BCz.

Because of the excellent efficiencies of the BII–BCz as a single host in D-EML white OLEDs, we further examined its performances in single-EML (S-EML) white devices. The device structure is the same as those of the monochrome devices, except for the EML with a configuration of BII–BCz:10 wt% FIrpic:0.6 wt% PO-01 (device WS). The blue and the yellow dopants are uniformly dispersed in the BII–BCz matrix and more efficient energy transfer is expected, leading to a warm white color emission. Device WS shows a very good color stability over a wide range of brightness, with a negligible CIE variation (ΔCIE) of only (0.009, 0.005) from 1000 to 10 000 cd m^–2^ (Fig. 6). It is worth noting that device WS reveals high performances with a maximum EQE and PE of 29.0% and 71.1 lm W^–1^, respectively, without any light out-coupling enhancement. The S-EML based device performs much better than the corresponding D-EML based devices, and this can be ascribed to the more direct energy transfer without a bilayer interface. Similar to the good performances of monochrome devices, device WS also exhibits mild efficiency decay as a function of luminance, and high efficiencies are still preserved with an EQE (PE) of 28.2% (58.2 lm W^–1^) at 1000 cd m^–2^. Since light is only detected from the forward-viewing direction, it is believed that the total energy emitted from a device is ∼1.7 times greater than that from the forward-viewing one.^45^ It is expected that the PE of device WS can ideally reach over 100 lm W^–1^ at 1000 cd m^–2^, which is comparable to that of commercial fluorescent tubes, representing one of the most efficient white OLEDs so far.^16^,^46^–^50^ To the best of our knowledge, this is the first single host based white PhOLED that can simultaneously realize EQE ≥25% at high brightness (≥1000 cd m^–2^) and extremely stable EL emission with ΔCIE < (0.01, 0.01) from 1000 to 10 000 cd m^–2^.

## Conclusions

To conclude, in this work we design a new D–σ–A system by using the sp^3^ carbon attached to a new BII moiety to link two electron-donating groups for the first time. High *E*_T_s, good thermal properties and suitable photophysical properties of the resulting BII–BCz and BII–TPA are demonstrated for their potential use as universal hosts for PhOLEDs. Both BII–BCz and BII–TPA show decent performances in blue, green, yellow, orange and red PhOLEDs with uniform simple device structures. The use of the new D–σ–A host materials allows us to obtain white OLEDs with a simple device structure without optical out-coupling enhancement. Among all the fabricated white devices, the S-EML white device based on BII–BCz as the single host exhibits state-of-the-art performances with a maximum EQE and PE of 29.0% and 71.1 lm W^–1^, respectively, with low efficiency roll-offs (EQE of 28.2% at 1000 cd m^–2^). Particularly, this device shows extremely stable color emission, with a ΔCIE of only (0.009, 0.005) from 1000 to 10 000 cd m^–2^. Our study may open a new pathway for designing efficient universal host materials for full-color PhOLED applications.

## Conflicts of interest

There are no conflicts to declare.

## Supplementary Material

Supplementary information
