# Supplementary material for: De novo design of D–σ–A molecules as universal hosts for monochrome and white phosphorescent organic light-emitting diodes
Source: Chem Sci. 2018 Mar 8;9(17):4062–70. doi: 10.1039/c8sc00282g (PMC5935058; doi:10.1039/c8sc00282g)
Supplement: Supplementary file 1 [file SC-009-C8SC00282G-s001.pdf]

Electronic Supplementary Material (ESI) for Chemical Science.
This journal is © The Royal Society of Chemistry 2018

Electronic Supplementary Information

De novo design of D-σ-A molecules as universal hosts for monochrome and white phosphorescence organic light-emitting diodes

Wen-Cheng Chen,[a] Yi Yuan,[b] Ze-Lin Zhu,[a] Zuo-Quan Jiang,[b] Shi-Jian Su,[c] Liang-Sheng Liao*[b] and Chun-Sing Lee*[a]

[a]*Center of Super-Diamond and Advanced Films (COSDAF) and Department of Chemistry, City University of Hong Kong, Hong Kong & City University of Hong Kong Shenzhen Research Institute, Shenzhen, Guangdong, PR China. E-mail: apcslee@cityu.edu.hk*
[b]*Jiangsu Key Laboratory for Carbon-Based Functional Materials & Devices, Institute of Functional Nano & Soft Materials (FUNSOM) & Collaborative Innovation Center of Suzhou Nano Science and Technology (Nano-CIC), Soochow University, Suzhou, 215123, PR China. E-mail: lsliao@suda.edu.cn*
[c]*State Key Laboratory of Luminescent Materials and Devices and Institute of Polymer Optoelectronic Materials and Devices, South China University of Technology, Guangzhou 510640, PR China.*

Contents

General information 1
Synthesis detail 1
Device fabrication and measurement 2
Thermal properties 3
Absorption and PL spectra of films 3
Solvent-dependent PL spectra 4
Optimal molecular configurations 4
Absorption of dopants and PL of hosts 5
Cyclic voltammetry 5
EL spectra of monochrome devices 6
Transient decay PL spectra 6
J-V-L characteristics of monochrome devices 7
Single carrier-only devices 8
EL spectra of white OLEDs 8
Reference 9

General information

^{1}H and ^{13}C NMR spectra were recorded on a Varian Unity Inova 400 spectrometer. Mass analyses were performed on an Applied Biosystems API-2000 liquid chromatography/mass spectrometry/mass spectrometry (LC/MS/MS) Triple-Q mass spectrometer with electrospray ionization source. Thermal weight change analysis was recorded on a thermal gravimetric analyzer (TGA Q50, TA Instruments). Temperature for 5% weight loss is used as the decomposition temperature. Glass transition temperatures were determined with a Perkin-Elmer DSC 7 differential scanning calorimetric at a heating rate of 10 °C minute^{-1} under a nitrogen atmosphere. UV-vis absorption spectra were acquired with an Agilent 8453 spectrophotometer. Photoluminescence spectra were measured on a Hitachi F-4500 Fluorescence spectrophotometer. Cyclic voltammetry was scanned on a CHI600 voltammetric analyzer equipped with a three-electrode system (platinum disk: working electrode, platinum wire: auxiliary electrode, Ag/AgCl: reference electrode). Ferrocene with an absolute highest occupied molecular orbital level of -4.80 eV was used as an internal standard. Nitrogen-saturated 0.1 mol L^{-1} tetrabutylammonium hexafluorophosphate CH_2Cl_2 (oxidation scan) or DMF (reduction scan) solution was used as the supporting electrolyte.

Synthesis detail

*11*H-benzo[4,5]imidazo[1,2-*a*]indol-11-one (BIO) was prepared according to the literature.[1]

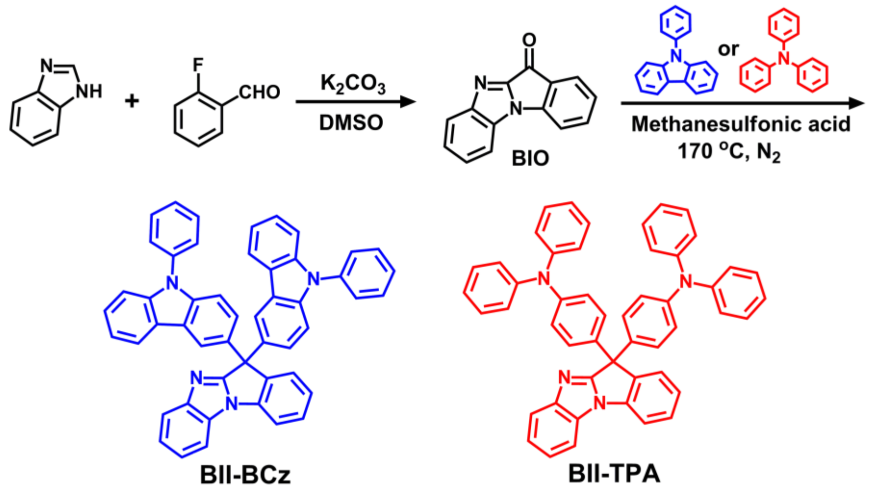

Scheme S1 Synthesis of BII-BCz and BII-TPA.

11,11-bis(9-phenyl-9*H*-carbazol-3-yl)-11*H*-benzo[4,5]imidazo[1,2-*a*]indole (BII-BCz): BIO (2 mmol, 0.44 g), 9-phenyl-9*H*-carbazole (40 mmol, 9.7 g) and methanesulfonic acid (1 mL) were mixed in a two-neck flask. The mixture was heated to 170 °C under a N_2 atmosphere. After 10 h reaction, the resulting mixture was cooled to room temperature, and then extracted with CH_2Cl_2. The extracted solution was washed with saturated $NaHCO_3$ aq., and dried by anhydrous $MgSO_4$. After evaporation of the solvent, the residue was purified via column chromatography. First, petroleum ether (PE) was used as eluent to separate the excessive non-polar triphenylamine, and then CH_2Cl_2/PE mixture (1:1, v/v) was used as eluent to give a white solid (0.86 g, 62.5%). ^{1}H NMR (400 MHz, DMSO-*d*6) δ [ppm]: 8.19 (d, J = 8.0 Hz, 1H), 8.15 (d, J = 1.8 Hz, 2H), 8.08 (d, J = 7.8 Hz, 1H), 8.03 (d, J = 7.8 Hz, 2H), 7.87 (d, J = 7.6 Hz, 1H), 7.78 (d, J = 8.1 Hz, 1H), 7.64 – 7.58 (m, 4H), 7.59 – 7.54 (m, 5H), 7.51 – 7.43 (m, 4H), 7.42 – 7.27 (m, 9H), 7.18 – 7.14 (m, 2H). ^{13}C NMR (151 MHz, $CDCl_3$) δ [ppm]: 165.23, 145.86, 141.55, 141.20, 140.05, 137.55, 134.73, 129.79, 129.60, 128.53, 127.68, 127.41, 126.97, 126.78, 125.94, 124.57, 123.23, 123.19, 122.68, 121.00, 120.53, 119.86, 119.83, 111.19, 110.67, 110.07, 109.73, 58.74. MS (ESI): *m/z* = 689.4 $[M + H]^+$.

4,4'-(11*H*-benzo[4,5]imidazo[1,2-*a*]indole-11,11-diyl)bis(N,N-diphenylaniline) (BII-TPA): Prepared as white powder (yield: 71.2%) in a similar manner as BII-BCz by using triphenylamine as the starting materials. ^{1}H NMR (400 MHz, DMSO-*d*6) δ [ppm]: 8.21 (d, J = 7.8 Hz, 1H), 8.07 (d, J = 7.8 Hz, 1H), 7.80 (d, J = 7.8 Hz, 1H), 7.70 (d, J = 7.6 Hz, 1H), 7.59 (t, J = 7.6 Hz, 1H), 7.48 – 7.41 (m, 1H), 7.37 (t, J = 7.6 Hz, 2H), 7.34 – 7.19 (m, 12H), 7.10 – 6.90 (m, 17H). ^{13}C NMR (151 MHz, $CDCl_3$) δ [ppm]: 163.63, 147.43, 146.90, 146.34, 139.57, 136.61, 135.73, 129.54, 128.94, 127.43, 124.83, 124.07, 123.41, 123.20, 122.73, 120.10, 111.76, 111.58, 56.59. MS (ESI): *m/z* = 693.6 $[M + H]^+$.

Device fabrication and measurement

Pre-cleaned indium tin oxide (ITO) coated glass substrates with a sheet resistance of 15 Ω/□ were used as transparent anodes. Before use, the substrates were treated a 20 min UV-ozone bath, and then immediately transferred into a deposition chamber with vacuum better than 10^{-7} Torr. Current density-voltage characteristics and electroluminescence spectra were recorded

with a Keithley 237 power source and a Spectrascan PR650 photometer, respectively. Device measurement was performed under an ambient condition.

Thermal properties

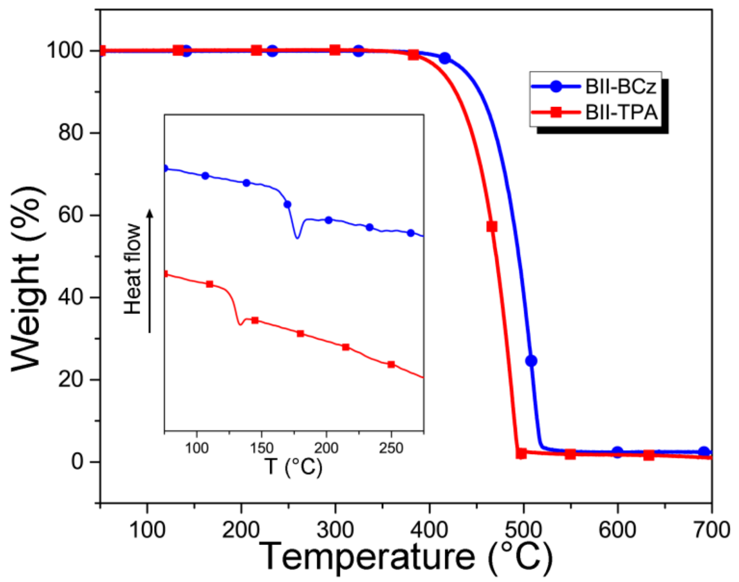

Fig. S1 TGA and DSC (inset) measurements of the new hosts.

Absorption and PL spectra of films

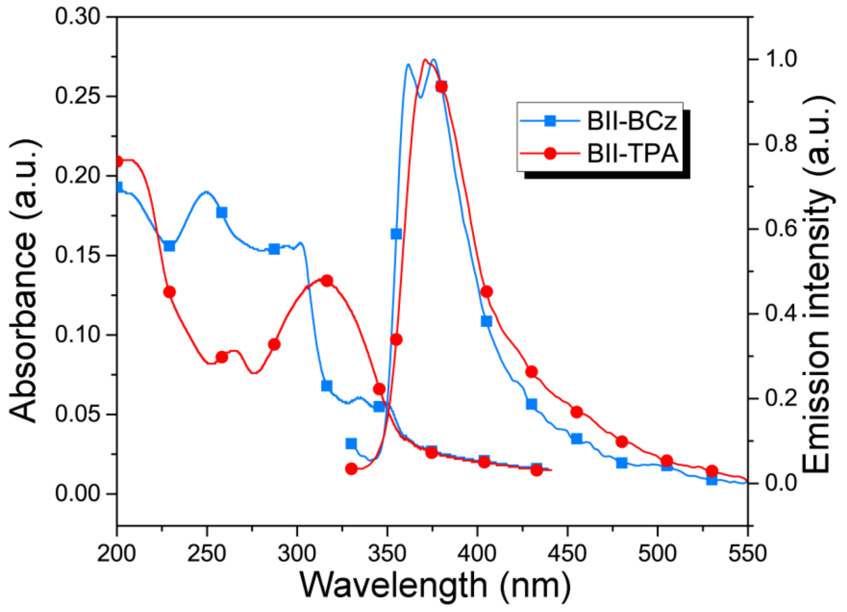

Fig. S2 Absorption and PL spectra of BII-BCz and BII-TPA in 30-nm films prepared on quartz substrates by thermal evaporation.

Solvent-dependent PL spectra

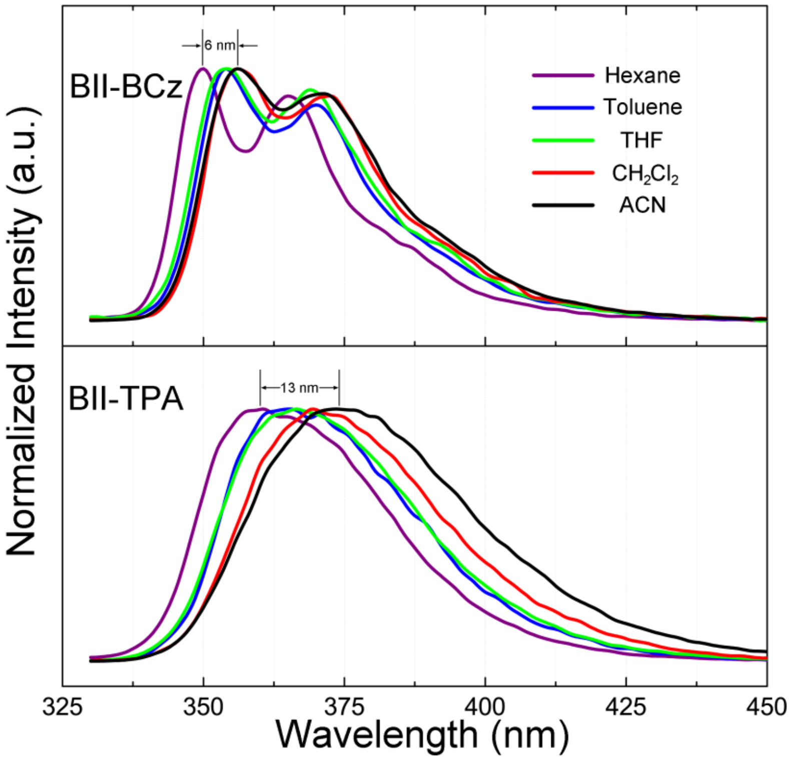

Fig. S3 PL spectra of BII-BCz and BII-TPA measured in different solvents (~10^{-6} mol L^{-1})

Optimal molecular configurations

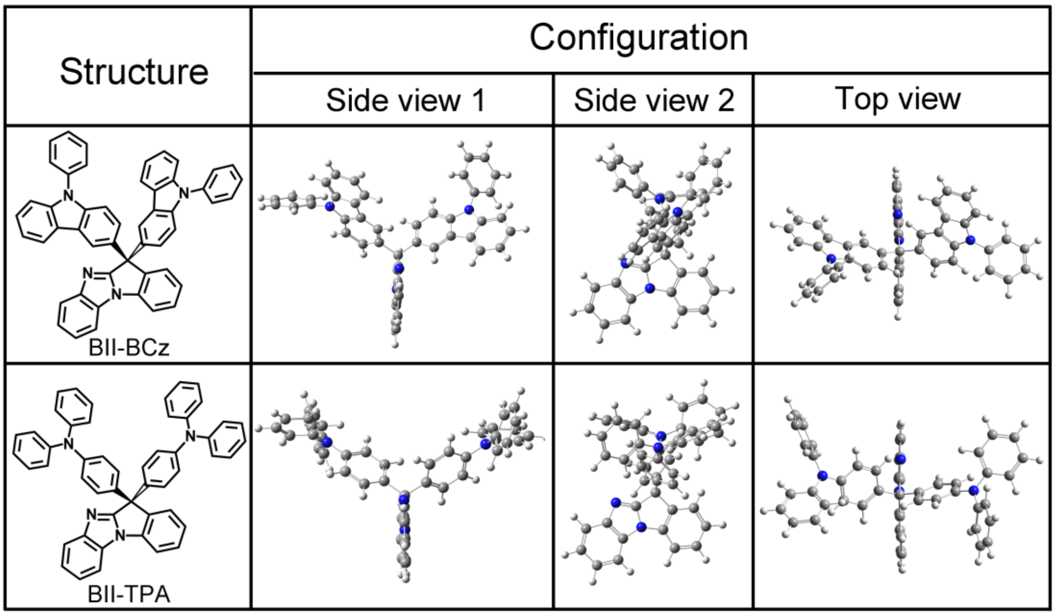

Fig. S4 Energy minimized molecular configurations of BII-BCz and BII-TPA.

Absorption of dopants and PL of hosts

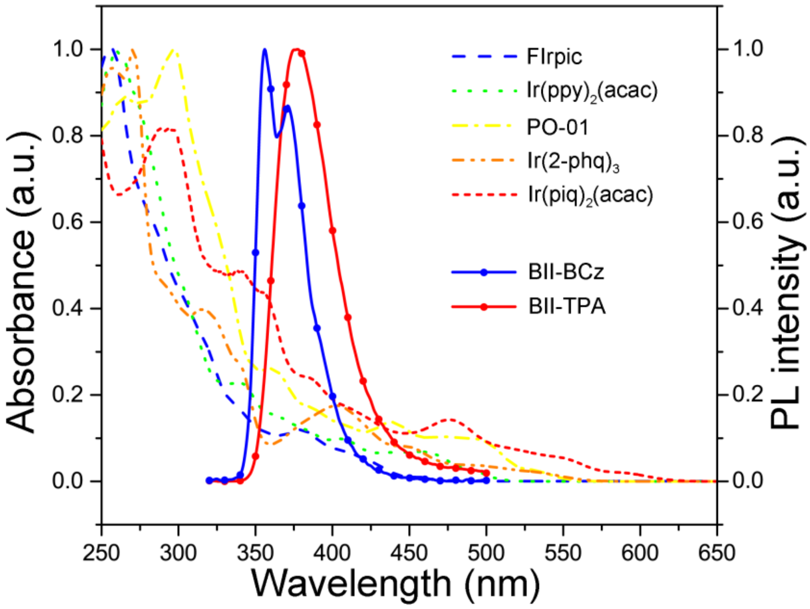

Fig. S5 Absorption spectra of the phosphorescence dopants (dash lines) and PL spectra of the hosts (solid lines) in CH_2Cl_2.

Cyclic voltammetry

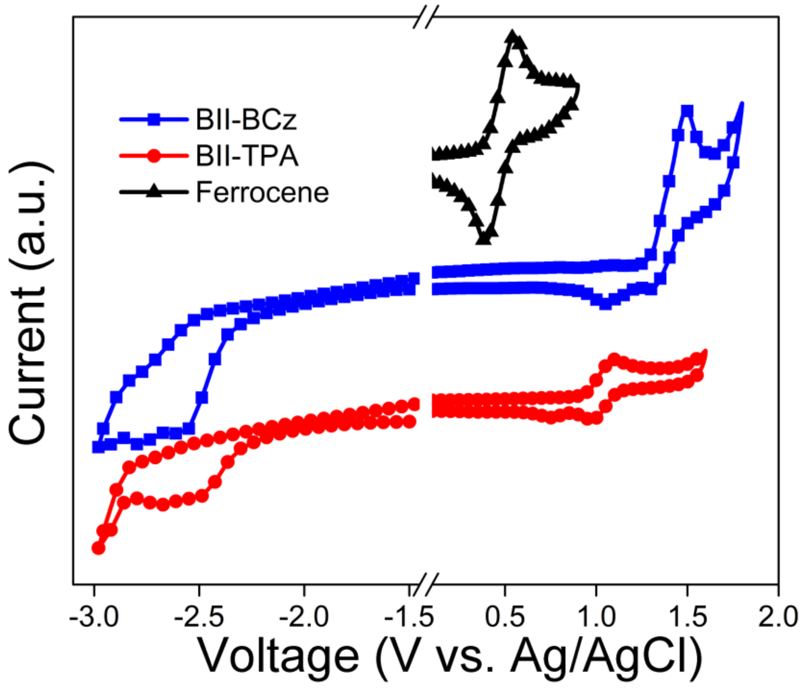

Fig. S6 Cyclic voltammetry of the new compounds; the HOMO/LUMO levels are estimated to be -2.03/-5.70 eV and -2.05/-5.34 eV for BII-BCz and BII-TPA, respectively.

EL spectra of monochrome devices

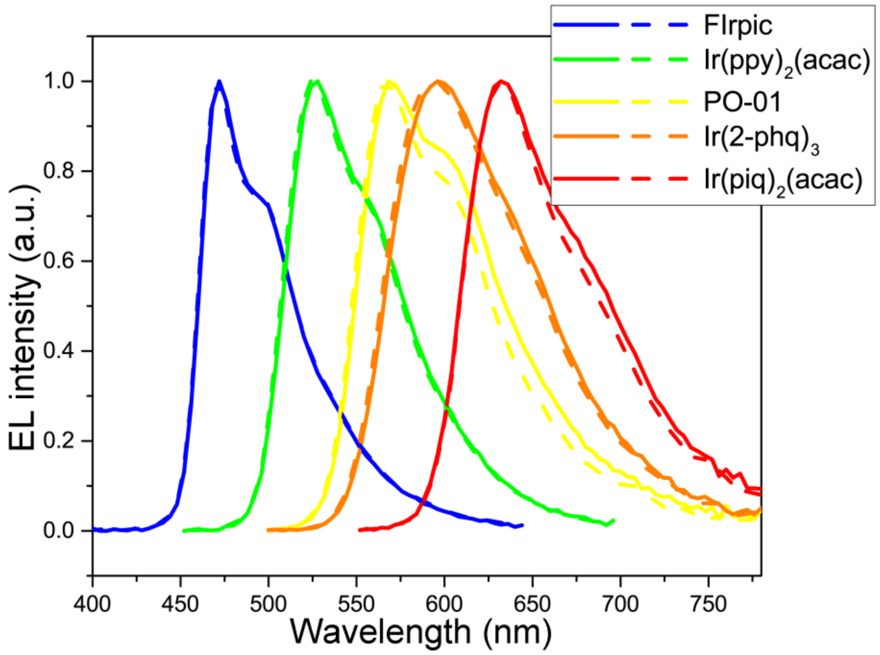

Fig. S7 EL spectra of the monochrome devices; solid and dash lines are for the BII-BCz and the BII-TPA based devices, respectively.

Transient decay PL spectra

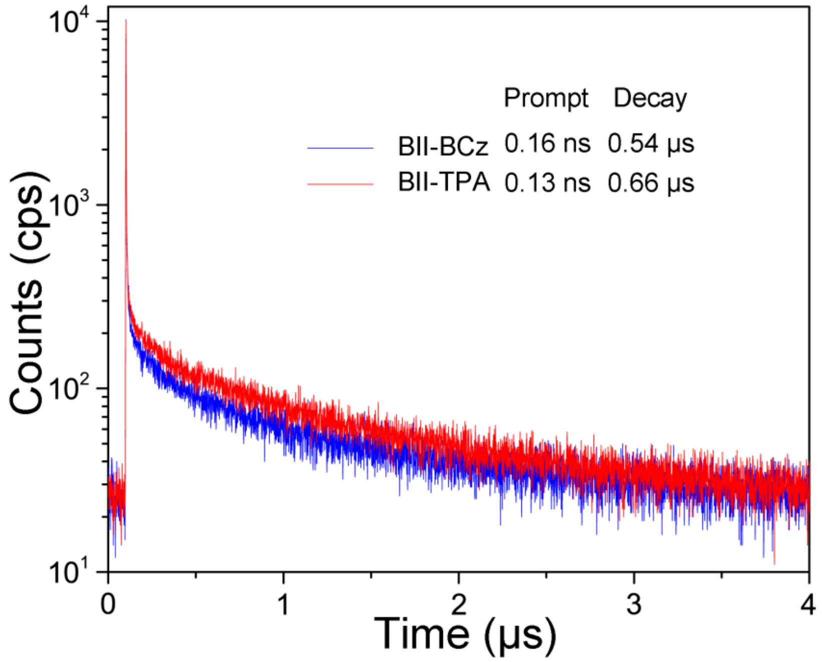

Fig. S8 Transient decay PL spectra of the host:10 wt% FIrpic doped films (30 nm) prepared on quartz substrates.

J-V-L characteristics of monochrome devices

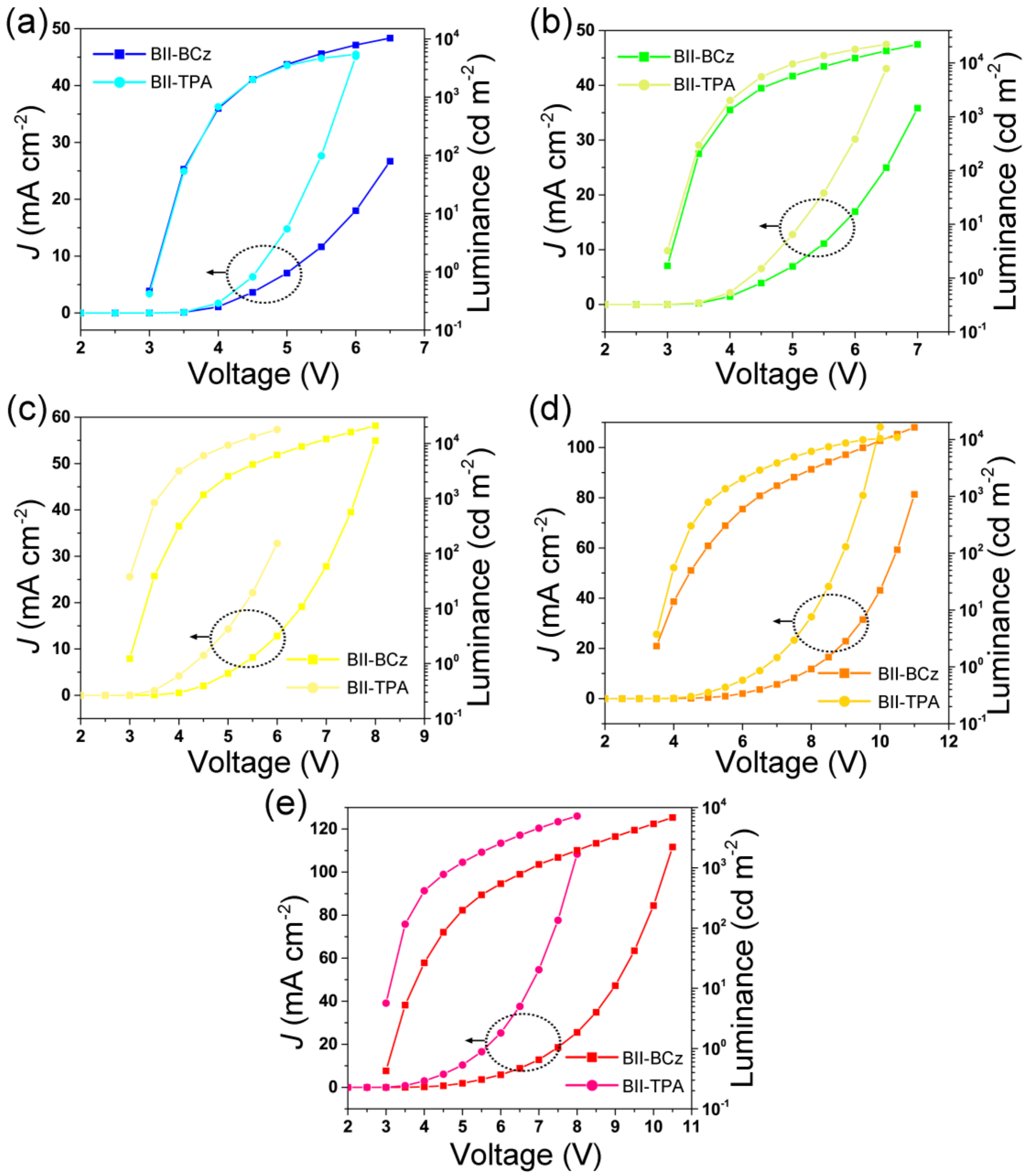

Fig. S9 *J-V-L* characteristics of the devices based on (a) FIrpic, (b) $Ir(ppy)_2(acac)$, (c) PO-01, (d) $Ir(2\text{-}phq)_3$ and (e) $Ir(piq)_2(acac)$ as dopants, respectively.

Single carrier-only devices

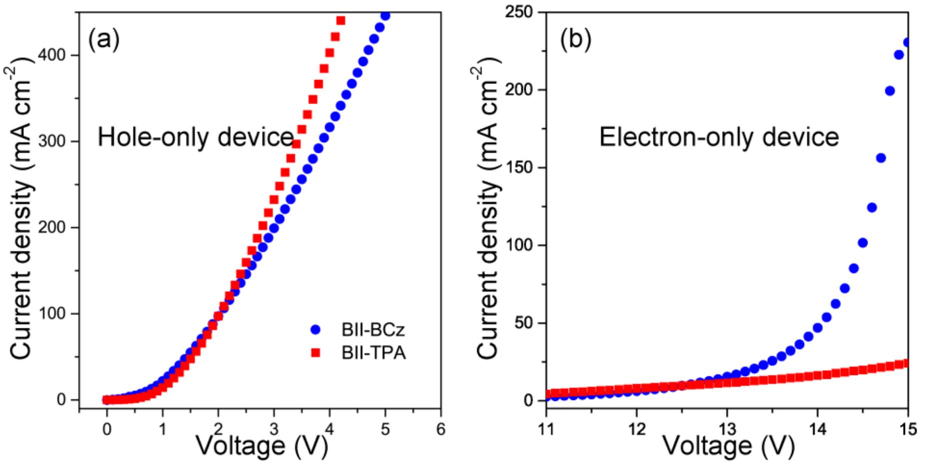

Fig. S10 *J-V* characteristics of (a) hole-only devices (HOD) and (b) electron-only devices (EOD). HOD: ITO/MoO_3 (10 nm)/BII-BCz or BII-TPA (50 nm)/MoO_3 (10 nm)/Al; EOD: ITO/TmPyPB (10 nm)/BII-BCz or BII-TPA (50 nm)/TmPyPB (10 nm)/LiF (1 nm)/Al.

EL spectra of white OLEDs

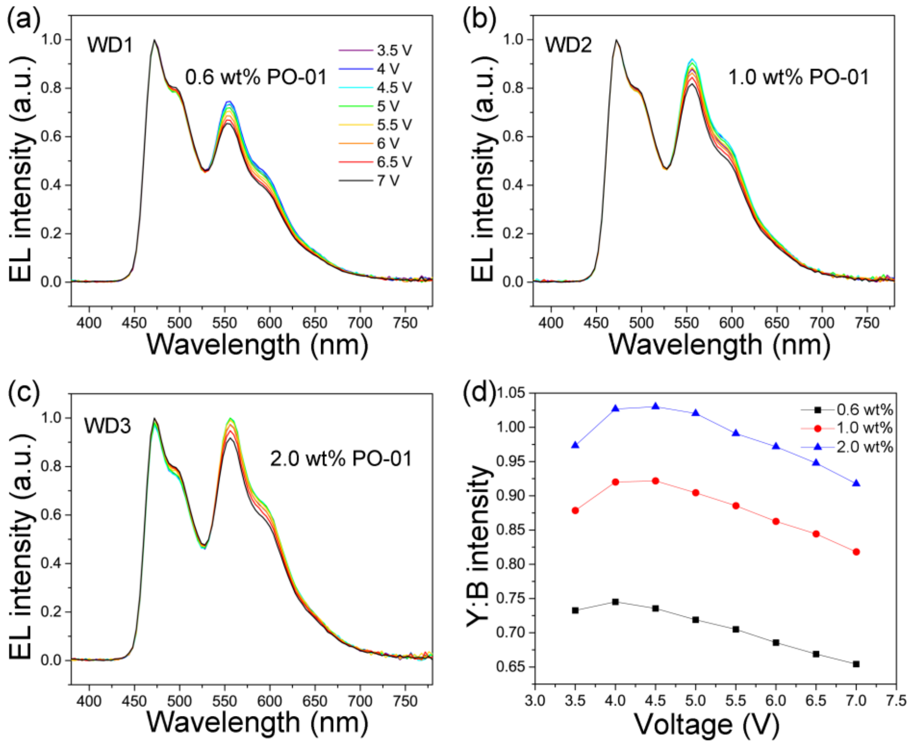

Fig. S11 Voltage-dependent EL spectra of the D-EML white OLEDs based on BII-BCz, with a PO-01 concentration of (a) 0.6 wt%, (b) 1.0 wt% and (c) 2.0 wt%, respectively. (d) Plots of the yellow to blue emission intensity as a function of voltage.

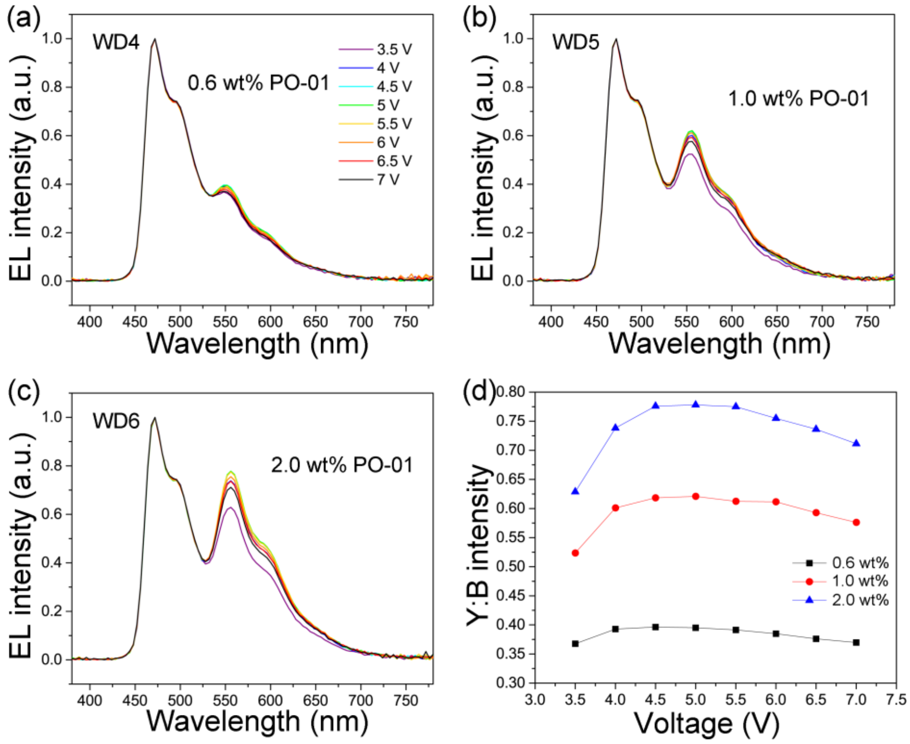

Fig. S12 Voltage-dependent EL spectra of the D-EML white OLEDs based on BII-TPA, with a PO-01 concentration of (a) 0.6 wt%, (b) 1.0 wt% and (c) 2.0 wt%, respectively. (d) Plots of the yellow to blue emission intensity as a function of voltage.

Reference

1 J. Rosevear and J. F. K. Wilshire, *Aust. J. Chem.*, 1991, **44**, 1097–1114.
